# Supplementary material for: Polyvalent Mannuronic Acid-Coated Gold Nanoparticles for Probing Multivalent Lectin–Glycan Interaction and Blocking Virus Infection
Source: Viruses. 2025 Jul 30;17(8):1066. doi: 10.3390/v17081066 (PMC12390540; doi:10.3390/v17081066)
Supplement: Supplementary file 1 [file viruses-17-01066-s001.zip › viruses-3705074-supplementary.pdf]

SUPPORTING INFORMATION (SI)

Polyvalent mannuronic acid-coated gold nanoparticles for probing multivalent lectin-glycan interactions and blocking virus infection

Rahman Basaran,[1,2] Darshita Budhadev,[1] Eleni Dimitriou,[3] Hannah S. Wootton,[3] Gavin J. Miller,[3] Amy Kempf,[4,5] Inga Nehlmeier,[4] Stefan Pöhlmann,[4,5] Yuan Guo,[6,] and Dejian Zhou[1,]**

[1] School of Chemistry and Astbury Centre for Structural Molecular Biology, University of Leeds, Leeds LS2 9JT, United Kingdom

[2] Department of Chemistry, Boğaziçi University, Istanbul, 34342, Türkiye.

[3] Centre for Glycoscience and School of Chemical and Physical Sciences, Keele University, Keele, Staffordshire, ST5 5BG, United Kingdom

[4] Infection Biology Unit, German Primate Center - Leibniz Institute for Primate Research, 37077, Göttingen, Germany.

[5] Faculty of Biology and Psychology, Georg-August-University Göttingen, 37073, Göttingen, Germany.

[6] School of Food Science and Nutrition, and Astbury Centre for Structural Molecular Biology, University of Leeds, Leeds LS2 9JT, United Kingdom

Contents

1. Instrument and Methods S2
2. Characterisation of N_3-EG_2-ManA S3-S7
3. Characterisation of LA-EG_2-ManA Ligand S7-S8
4. Determination of Glycan Valency on GNP-ManA S9-S10
5. Fluorescence Spectra of DC-SIGN/R and GNP-ManA + DC-SIGN/R S11
6. Viral Inhibition Data S12
7. Cytotoxicity Test S13

1. Instrument and Methods

Unless otherwise stated, all reagents used in the following experiments were bought commercially from Acros Organics, Alfa Aesar, Biosynth, Fisher Scientific, Fluorochem, Sigma Aldrich, TCI chemicals, and Thermo Scientific and were used without further purification. All moisture-sensitive reactions were performed in oven-dried glassware under an atmosphere of N_2 either with a Schlenk line or an N_2 filled balloon. Solvents were dried and stored under N_2 in Young's flasks over 4 Å molecular sieves. For reactions that required heating, DrySyn heating blocks were used as the heat source. Evaporations were carried out at reduced pressure using a Bruker rotary evaporator and a Virtis Benchtop K freeze dryer. Column chromatography was performed using silica gel 60 A, and the progress of the reactions was monitored by thin layer chromatography, TLC, analysis on aluminium sheets pre-coated 0.25 mm 60 F_{254} silica gel plates (Merck) and eluent systems outlined in the respective experiments. The polar lipoic acid-sugar derivatives were purified by size exclusion chromatography via Biogel P2 column using 20 mM ammonium formate as an eluent to yield the desired pure product. Visualisation was achieved using UV light (λ = 254 nm), and 10% H_2SO_4 in EtOH followed by heating. Flash column chromatography was performed using silica gel [high purity grade, 60 Å pore size, 40-63 μm particle size] or *via* automation using a Buchi Reveleris X2 system.

All ^{1}H and ^{13}C NMR spectra were recorded on a Bruker AV4 NEO-500 (500 MHz for ^{1}H, 125 MHz for ^{13}C) and a Bruker AV3HD-400 (400 MHz for ^{1}H, 100 MHz for ^{13}C) spectrometer in appropriate undeuterated solvents. All chemical shifts (δs) are denoted in parts per million (ppm) calibrated using residual undeuterated solvents as internal references ($CDCl_3$: δ ^{1}H = 7.26 ppm, δ ^{13}C = 77.16 ppm; CD_3OD: δ ^{1}H = 3.31 ppm, δ ^{13}C = 49.15 ppm; D_2O: δ ^{1}H = 4.80 ppm). The coupling constants (*J*) are in parentheses and expressed in Hertz, Hz, and the peak patterns are indicated with the following abbreviations: s = singlet, d = doublet, t = triplet, q = quartet, m = multiplet, br = broad, dd = doublet of doublets, dt = doublet of triplets, td = triplet of doublets, dq= doublet of quartets, qt = quartet of triplets, ddd = doublet of doublet of doublets, dtd = doublet of triplet of doublets. For sugar synthesis, HRMS (High-resolution mass spectra) data were recorded on a Thermo Scientific LTQ Orbitrap XL at the ESPRC National Mass Spectrometry Facility at Swansea University. Optical rotations were recorded on a Bellingham + Stanley ADP430 (specific rotation, tube length: 50 mm, concentrations in g per 100 mL). For produced ligand analysis and protein studies, HRMS data were obtained on a Bruker Daltonics MicroTOF mass spectrometer, and deconvoluted mass values (*m/z*) are

reported in Daltons, and protein labelling efficiency was also calculated from the ratio of the integral of the labelled protein HR-MS peak to the sum of that of the labelled and unlabelled protein peaks. The mass spectrometry data were also collected using a Bruker HCT Ultra coupled to Ultimate 3000 HPLC (Thermo Scientific). Methanol was used as a solvent to ionise the products; this provides a high-precision detection of ionised samples by classifying the resulting ions by vacuum with respect to mass-charge ratios (*m/z*). UV-vis absorption spectra were recorded on either a Cary 60 UV-vis spectrophotometer (Agilent Technologies) over 200-800 nm using 1 mL quartz cuvette with an optical path length of 1 cm or on a Nanodrop 2000 spectrophotometer (Thermo Scientific) over the range of 200-800 nm using one drop of the solution with an optical path length of 1 mm.

1. Synthesis of [2-(2-azidoethoxy)ethoxy] α-D-mannopyranosiduronic acid [30-32]

1.1. 2,3,4,5-Tetra-*O*-acetyl-α-D-[2-(2-azidoethoxy)ethoxy]mannopyranoside (2)

(A) ^{1}H NMR (400 MHz, $CDCl_3$)

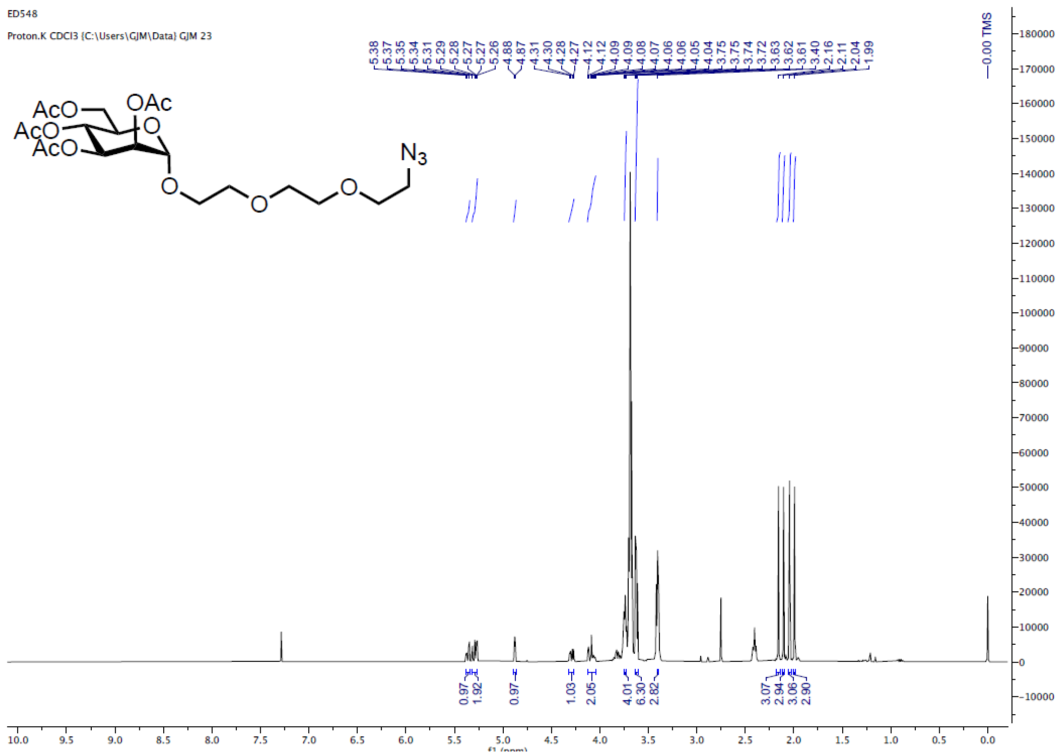

(B) ^{13}C NMR (101 MHz, $CDCl_3$)

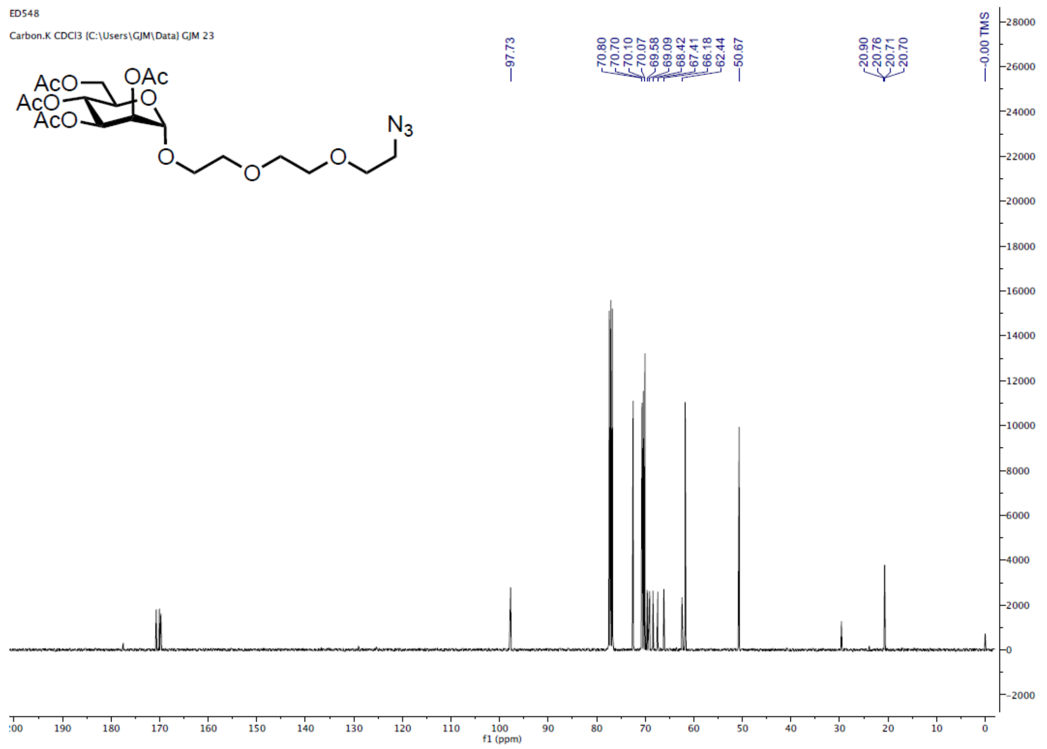

Figure S1. ^{1}H-NMR (A) and ^{13}C-NMR (B) spectrum of Compound 2.

1.2. **2,3,4,5-Tetra-*O*-hydroxy-α-D-[2-(2-azidoethoxy)ethoxy]mannopyranoside (3)**

(A) ^{1}**H NMR (400 MHz,** $CDCl_3$**)**

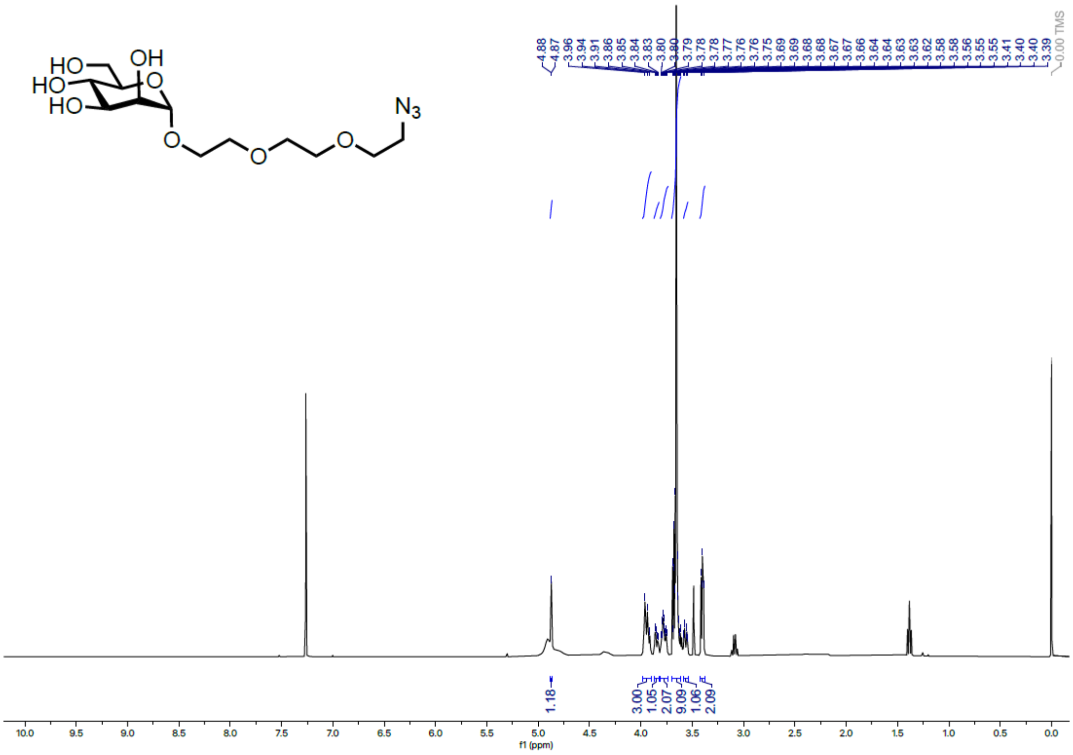

(B) ^{13}C NMR (101 MHz, $CDCl_3$)

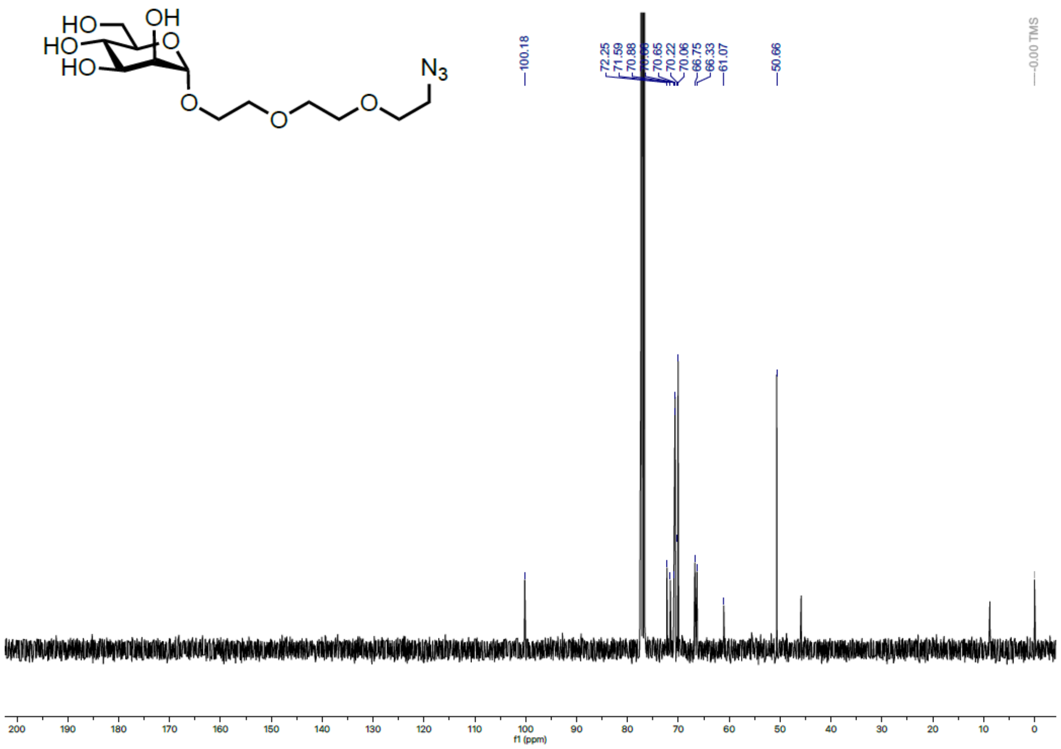

Figure S2. ^{1}H-NMR (A) and ^{13}C-NMR (B) spectrum of Compound 3.

1.3. [2-(2-azidoethoxy)ethoxy] α-D-mannopyranosiduronic acid (4)

(A) ^{1}H NMR (400 MHz, $CDCl_3$)

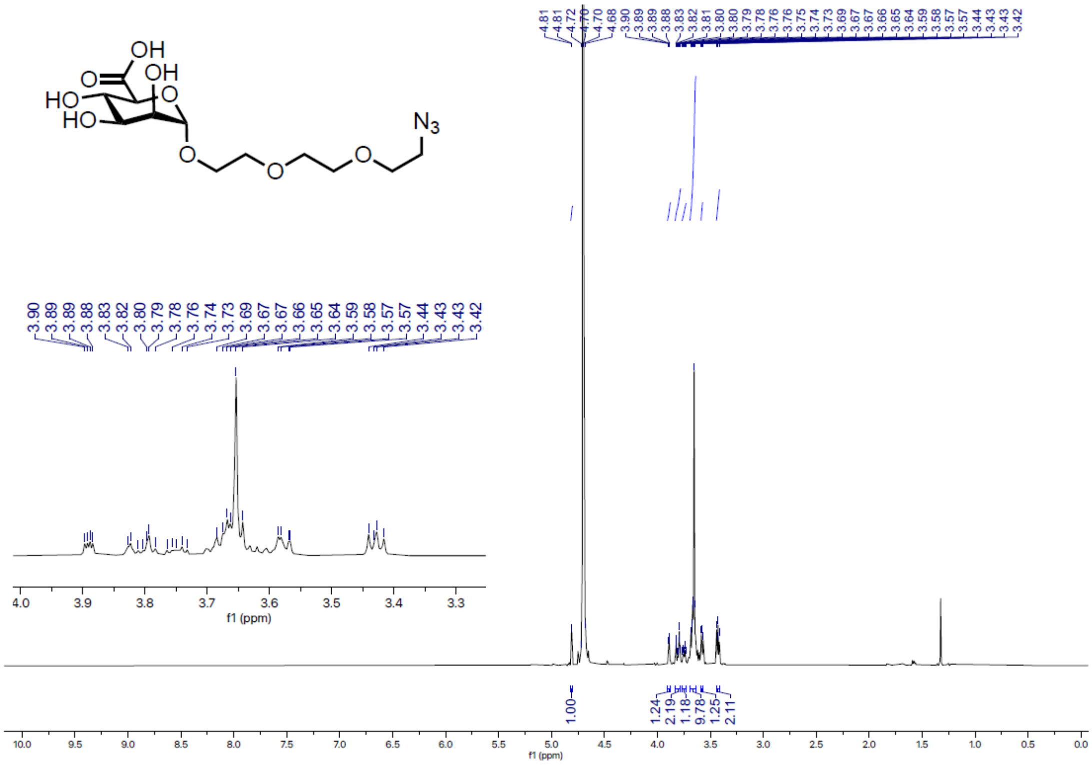

(B) ^{13}C NMR (101 MHz, $CDCl_3$)

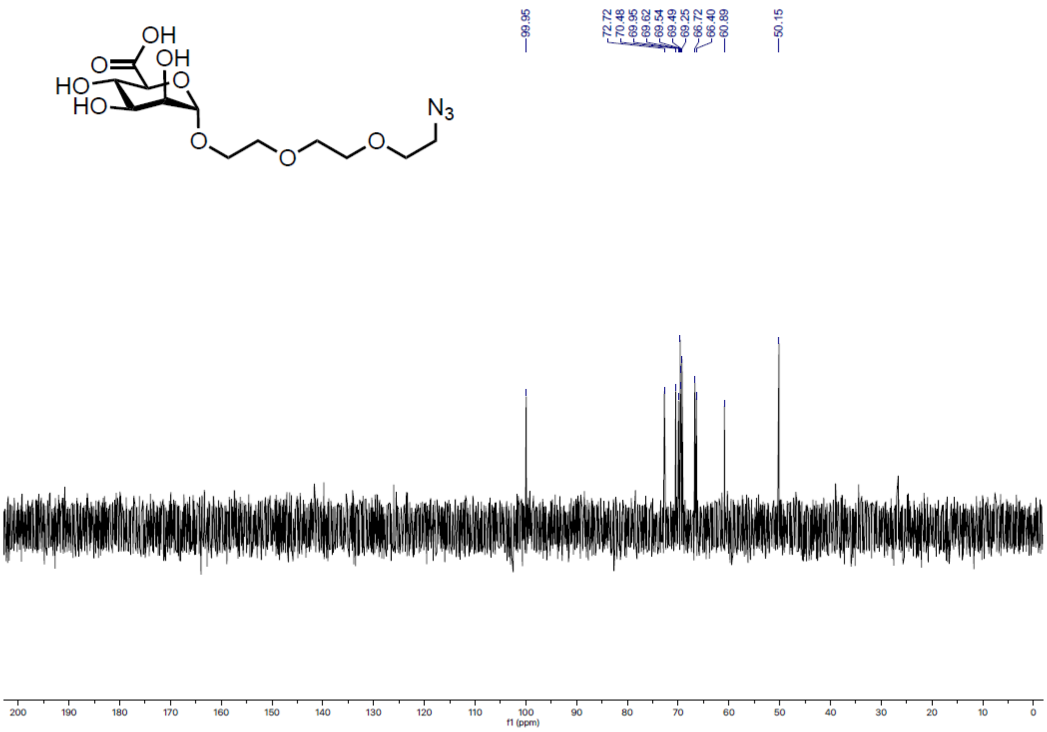

(C) HSQC-NMR

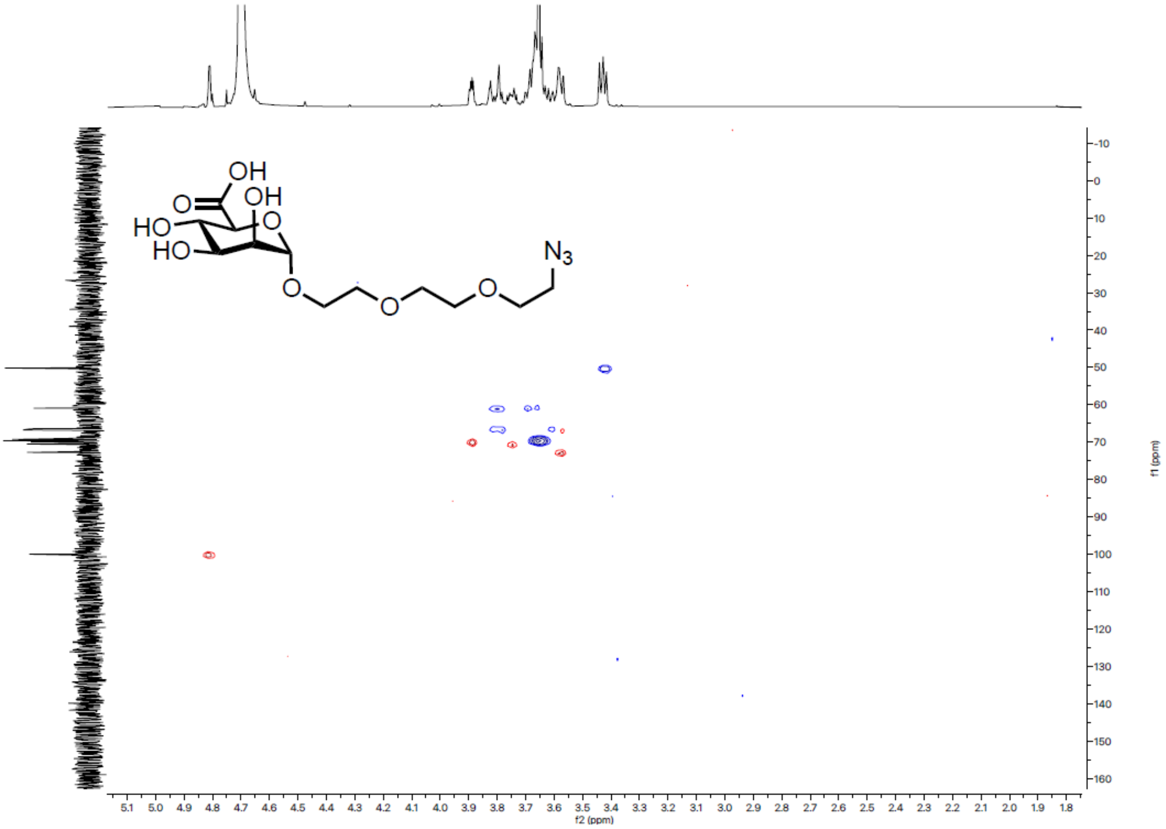

(D) COSY-NMR

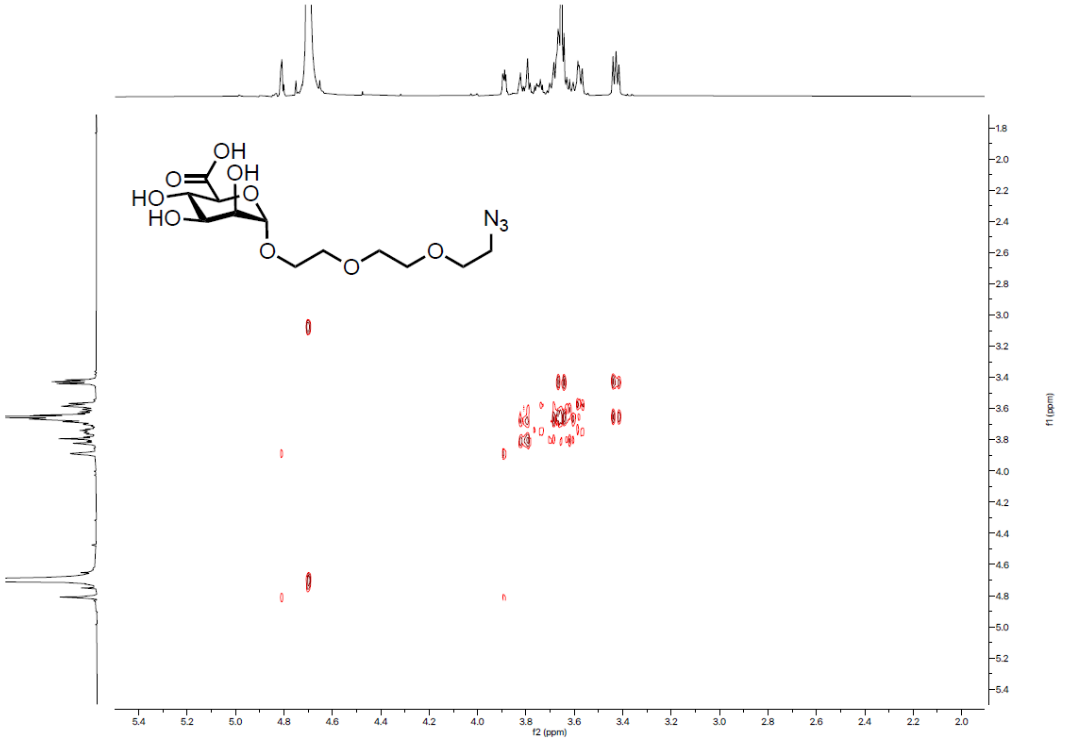

Figure S3. ^{1}H-NMR (A), ^{13}C-NMR (B), HSQC-NMR (C), and COSY-NMR (D) spectrum of Compound 4.

2. Synthesis of the LA-EG$_2$-ManA (5)

(A) ^{1}H-NMR of LA-EG$_2$-ManA

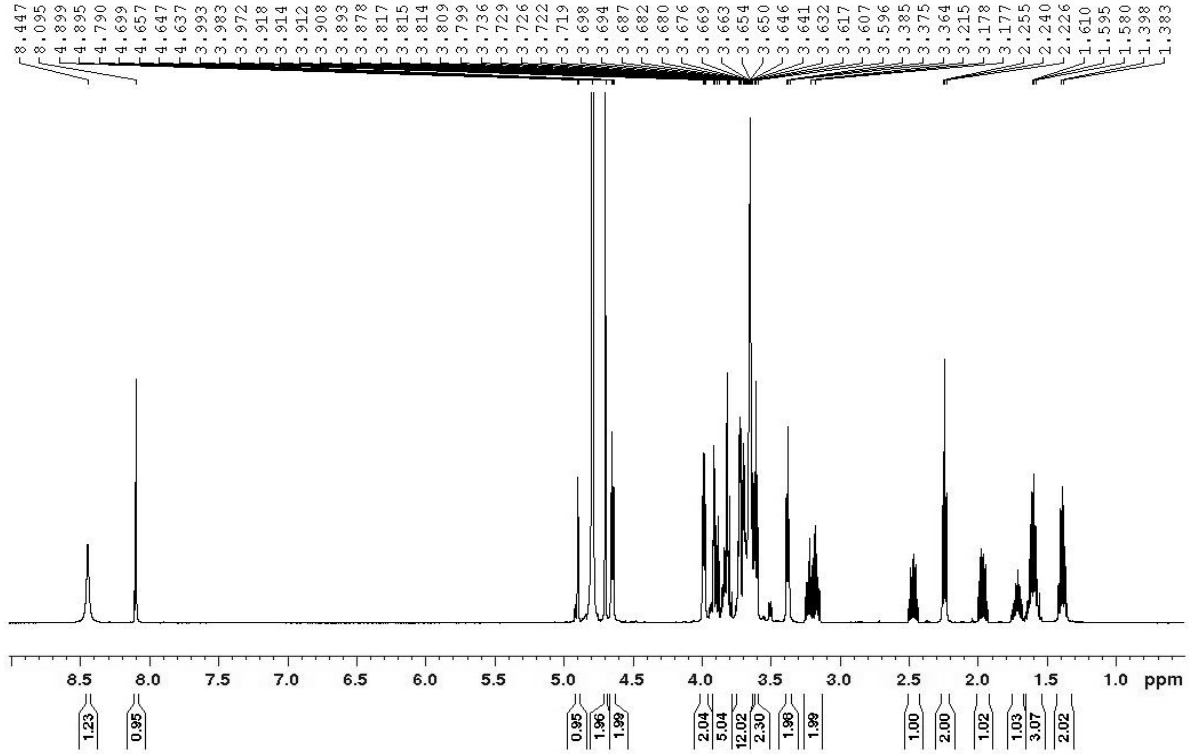

(B) ^{13}C-NMR of LA-EG$_2$-ManA

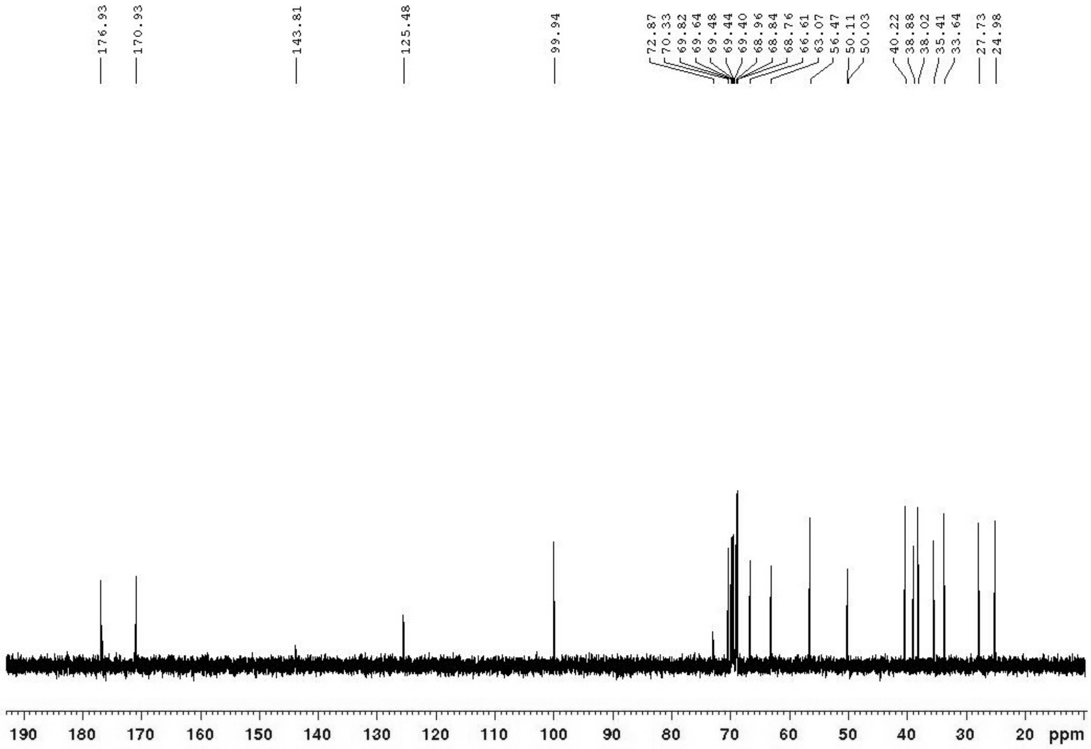

(C) LC-MS of LA-EG$_2$-ManA

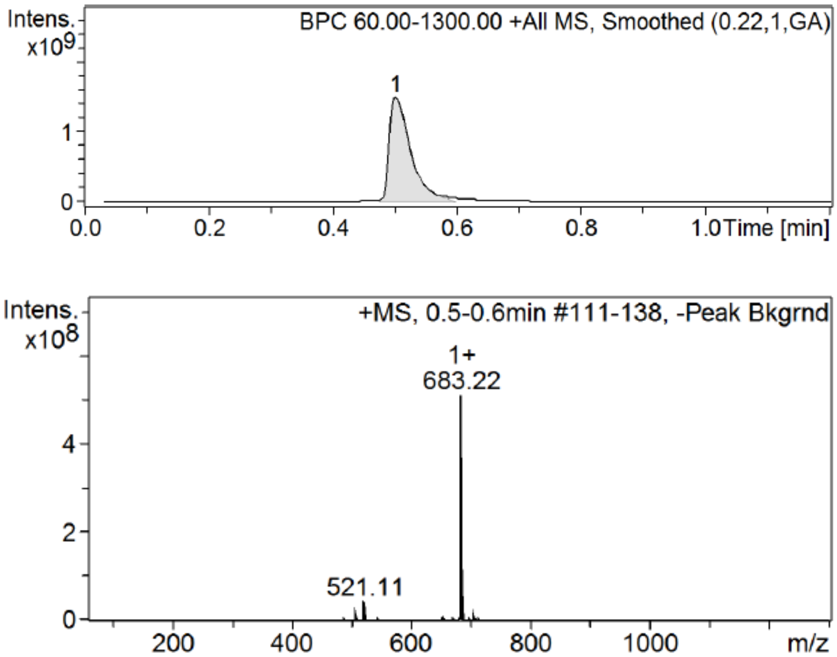

Figure S4. ^{1}H-NMR (A), ^{13}C-NMR (B) and LC-MS (C) spectrum of final LA-EG$_2$-ManA ligand.

3. Determination of Glycan Valency on GNP-ManA[14,29]

The supernatants and washing though liquids collected from GNP-ManA purification were used to determine the amount of unbounded free ligands using the phenol-sulfuric acid method for carbohydrate quantification.[39] 80 μL of 5% phenol solution and 400 μL of concentrated H_2SO_4 were added to a series of glycan ligand solutions in H_2O (80 μL) containing 2.0-20 μg of LA-EG_2-ManA ligand. The resulting mixtures were vortexed and then allowed to stand at RT for 30 mins. Their absorbance at 490 nm (A_{490}) were then recorded against a black water control. The A_{490}-concentration relationship was plotted by linear function to yield a calibration curve: Y = (0.03415 ± 0.0007) X (X in μM^{-1}).

The supernatants and washing-through filtrates were combined, freeze-dried, and then re-dissolved in 1.40 mL of pure water. 25 μL of each solution was diluted with water to a final volume of 125 μL. This solution was then mixed with 125 μL of 5% phenol and 625 μL of H_2SO_4 and incubated at RT for 30 mins. The absorbance of the mixture was recorded at 490 nm, and the dilution factors were corrected to calculate the total amount of unconjugated glycan ligand. The measurements were done in duplicate for each sample. The LA-EG_2-ManA ligand amount difference between that added and that remained unbound after GNP conjugation was counted as LA-EG_2-ManA ligands that have bound to the GNP. The average number of LA-EG_2-ManA ligands conjugated to each GNP were found ~530 for G5-ManA and ~1800 for G13-ManA.

4.1. Calculation of the average inter-glycan distance on GNPs[14,19,21]

The average inter-glycan distance (*d*) of G5/13-EG_2-ManA were calculated by using the D_h values and corresponding glycan valencies, based on the method reported by Hill *et al.*[49] For a GNP with a radius of r (equals to half of the hydrodynamic diameter of the GNP-glycan: $r = 1/2\ D_h$) and covered with N ligand, the footprint of each glycan (k) onto the GNP surface was calculated by the following equation:

$$k = \frac{4\pi r^2}{N}$$

Where r is hydrodynamic radius of the GNP-DiMan measured by DLS, where $r = 11.7/2 = 5.85$ nm for G5-ManA and $21.8/2 = 10.9$ nm for G13-ManA. The average deflection angle of each ligand on the GNP surface (θ, in degrees) was calculated via the equation below.

$$\theta = \frac{2 \times 180\, x \sqrt{\frac{k}{\pi}}}{r\pi}$$

The estimated θ for G5-ManA and G13-ManA (*N* = 530 and 1800) were 9.96^{o} and 5.6^{o}, respectively. By using these data, the inter-glycan distance on the GNP surface (*X*) was then calculated via the following equation. The results were given in **Table S1** below.

$$X = 2\, r\, \sin\left(\frac{\theta}{2}\right)$$

The average inter-glycan distances were estimated as ~1.01 and ~1.08 for G5-ManA and G13-ManA, respectively. These values match well the majority inter-glycan sequon spaces (*e.g.* 0.7−1.3 nm) found on the HIV surface glycoprotein gp120 trimer.[38] The comparable inter-glycan distances between Gx-ManA and HIV gp120 trimer suggested that the Gx-ManA conjugates are good mimics of viral glycoproteins to probe their interactions with DC-SIGN/R receptors.

Table S1. Summary of the chemical & physical parameters of G5/13-ManA conjugates.

**GNP-glycan**	**Glycan valency**	D_h **(nm)**	**Glycan footprint on GNP surface (nm^2)**	**Inter-glycan spacing (*d*) in nm**
**G5-ManA**	530 ± 48	11.7 nm	0.81	1.01 nm
**G13-ManA**	1800 ± 48	21.8 nm	0.80	1.08 nm

5. Fluorescence Spectra of DC-SIGN/R and GNP-ManA binding with DC-SIGN/R

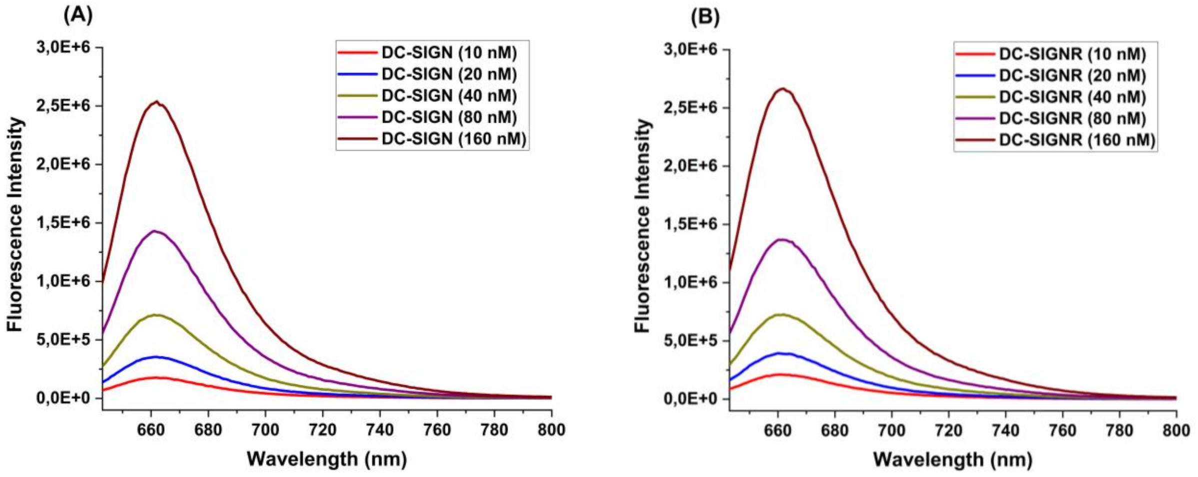

Figure S5. The fluorescence spectra of Atto-643 labeled DC-SIGN (**A**) and DC-SIGNR (**B**) only at varying concentrations (10 -160 nM).

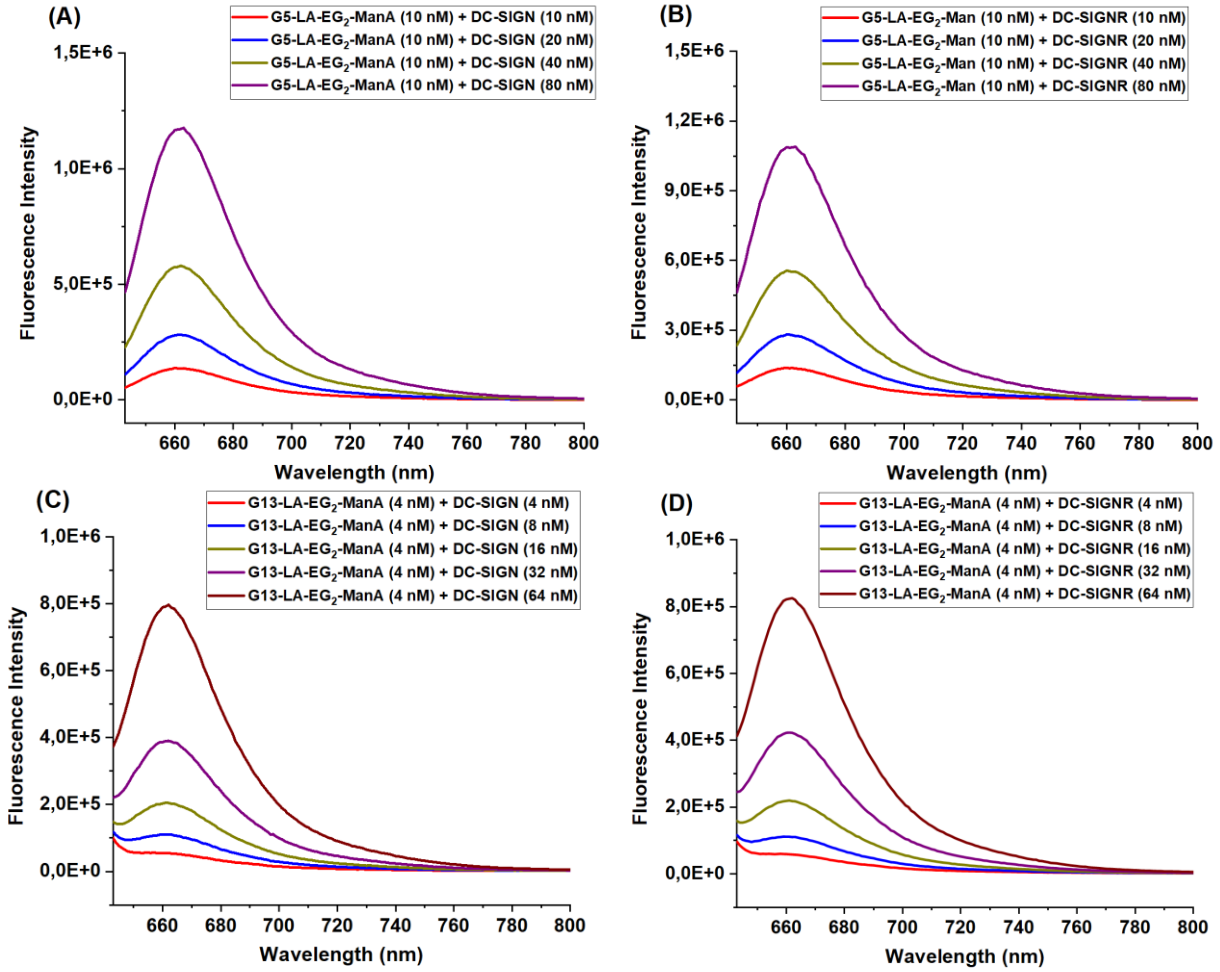

Figure S6. The fluorescence spectra of (A) G5-ManA+DC-SIGN; (B) G5-ManA+DC-SIGNR; (C) G13-ManA+DC-SIGN; and (D) G13-ManA+DC-SIGNR. The concentration of Gx-ManA was fixed at 10 nM (x = 5) or 4 nM (x = 13), while the concentration of DC-SIGN/R was varied to make samples of varying DC-SIGN/R: Gx-ManA molar ratios. Each concentration was done in duplicate and their average fluorescence spectra was used for QE calculation.

7. Viral Inhibition Data

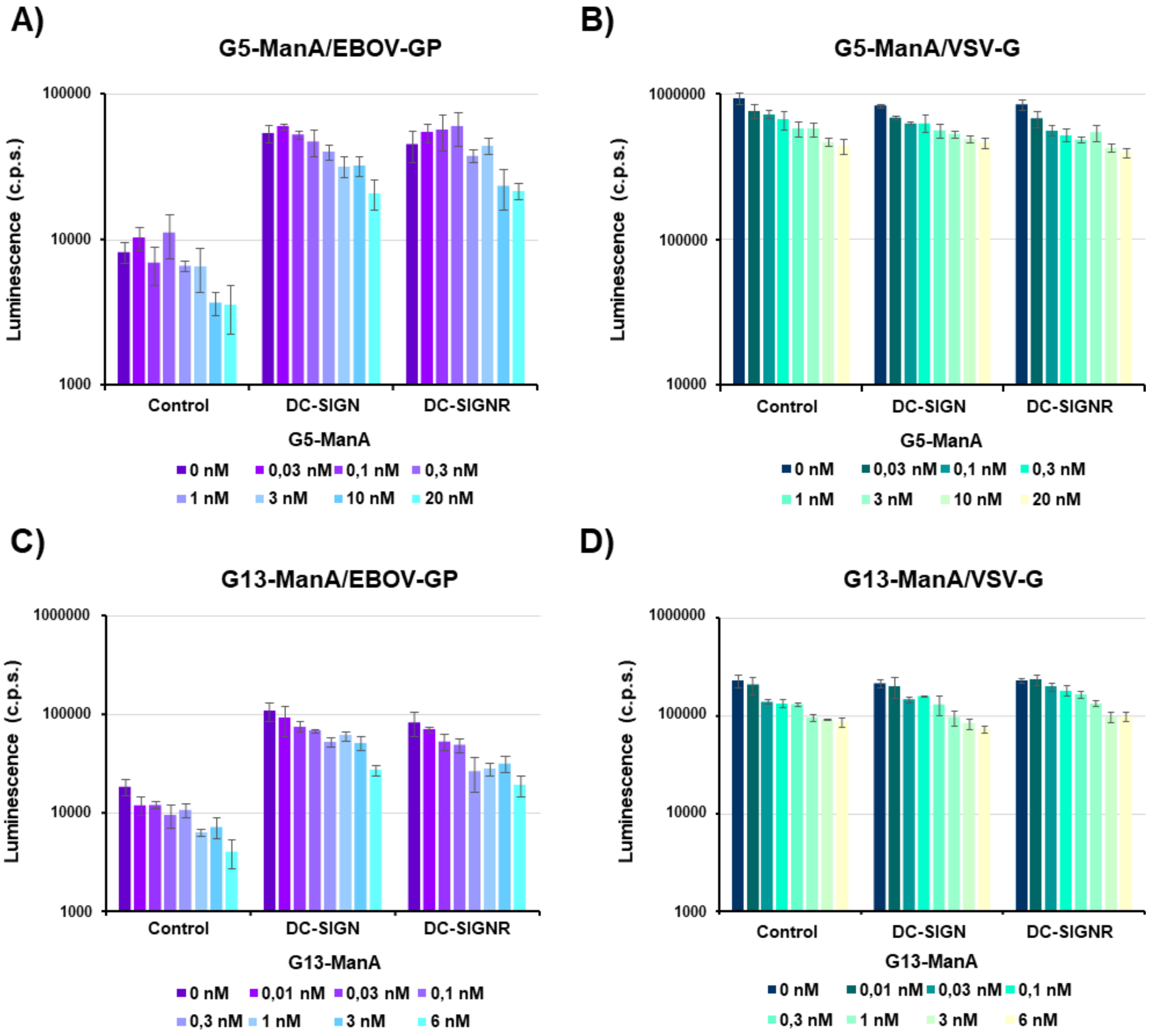

Figure S7. Cellular luciferase activities of 293T cells transfected to express DC-SIGN or DC-SIGNR or control transfected with empty vector (control) were pre-treated with the indicated concentrations of G5-ManA (**A, B**) and G13-ManA (**C, D**), and then inoculated with VSV particles encoding the luciferase gene and harboring EBOV-GP (**A, C**) or as control, VSV-G (**B, D**). The results of a single experiment performed with technical quadruplicates are shown. Error bars indicate standard deviations. Similar results were obtained in a separate experiment.

8. G5-ManA Cytotoxicity Test

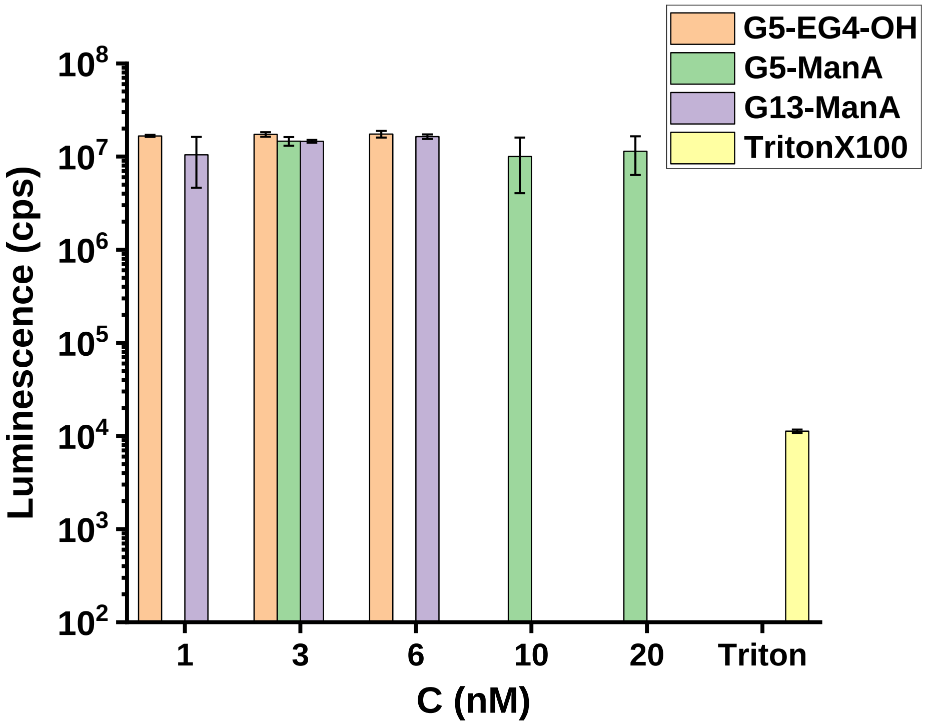

Figure S8. Evaluation of Gx-ManA cytotoxicity toward 293T cells measured by Cell Titer-Glo Assay. Treatment of the cells with the detergent Triton X100 served a positive control for cytotoxicity. No dose-dependent reduction of cell viability (indicated by luminescence) was observed for Gx-ManA at the highest concentrations used in antiviral tests, suggesting no measurable cytotoxicity. In fact, their cell viabilities are comparable to that treated with G5-EG_4-OH (G5 coated with LA-EG_4-OH ligand and shown to be noncytotoxic, see Ning et al., *JACS Au*, 2024, 4, 3295). The results of a single experiment performed with technical quadruplicates are shown. Error bars indicate standard deviations.
